# Automatic coronary artery plaque thickness comparison between baseline and follow‐up CCTA images

**DOI:** 10.1002/mp.13993

**Published:** 2020-01-20

**Authors:** Qing Cao, Alexander Broersen, Pieter H. Kitslaar, Mingyuan Yuan, Boudewijn P. F. Lelieveldt, Jouke Dijkstra

**Affiliations:** ^1^ Division of Image Processing Department of Radiology Leiden University Medical Center PO Box 9600 2300 RC Leiden The Netherlands; ^2^ Medis Medical Imaging System BV Leiden The Netherlands; ^3^ Zhoupu Hospital Shanghai University of medicine & health Science Shanghai China

**Keywords:** baseline and follow‐up, coronary arteries, coronary computed tomography angiography, plaque thickness, visualization

## Abstract

**Purpose:**

Currently, coronary plaque changes are manually compared between a baseline and follow‐up coronary computed tomography angiography (CCTA) images for long‐term coronary plaque development investigation. We propose an automatic method to measure the plaque thickness change over time.

**Methods:**

We model the lumen and vessel wall for both the baseline coronary artery tree (*CAT‐BL*) and follow‐up coronary artery tree (*CAT‐FU*) as smooth three‐dimensional (3D) surfaces using a subdivision fitting scheme with the same coarse meshes by which the correspondence among these surface points is generated. Specifically, a rigid point set registration is used to transform the coarse mesh from the *CAT‐FU* to *CAT‐BL.* The plaque thickness and the thickness difference is calculated as the distance between corresponding surface points. To evaluate the registration accuracy, the average distance between manually defined markers on clinical scans is calculated. Artificial *CAT‐BL* and *CAT‐FU* pairs were created to simulate the plaque decrease and increase over time.

**Results:**

For 116 pairs of markers from nine clinical scans, the average marker distance after registration was 0.95 ± 0.98 mm (two times the voxel size). On the 10 artificial pairs of datasets, the proposed method successfully located the plaque changes. The average of the calculated plaque thickness difference is the same as the corresponding created value (standard deviation ± 0.1 mm).

**Conclusions:**

The proposed method automatically calculates local coronary plaque thickness differences over time and can be used for 3D visualization of plaque differences. The analysis and reporting of coronary plaque progression and regression will benefit from an automatic plaque thickness comparison.

## 1 Introduction

Coronary artery disease (CAD) is still one of the leading causes of death worldwide^1^ and is caused by the buildup of plaque in the walls of the coronary arteries that supply blood to the heart muscle. To diagnose suspected CAD, coronary computed tomography angiography (CCTA) has been widely used. For the assessment of coronary artery plaques on CCTA images, different parameters can be measured, such as the minimum lumen diameter, minimum lumen area, and plaque volume which are calculated per coronary segment, per plaque, or even per patient.^2^, ^3^ Just lumen diameter assessment may lead to misinterpretation due to an irregular lumen shape caused by the plaque while area or volume assessment ignore local details. Furthermore, measurements based on the lumen do not consider the situation that the lumen remains the same while the plaque buildup enlarges the vessel wall (positive remodeling).

**Figure 1:**
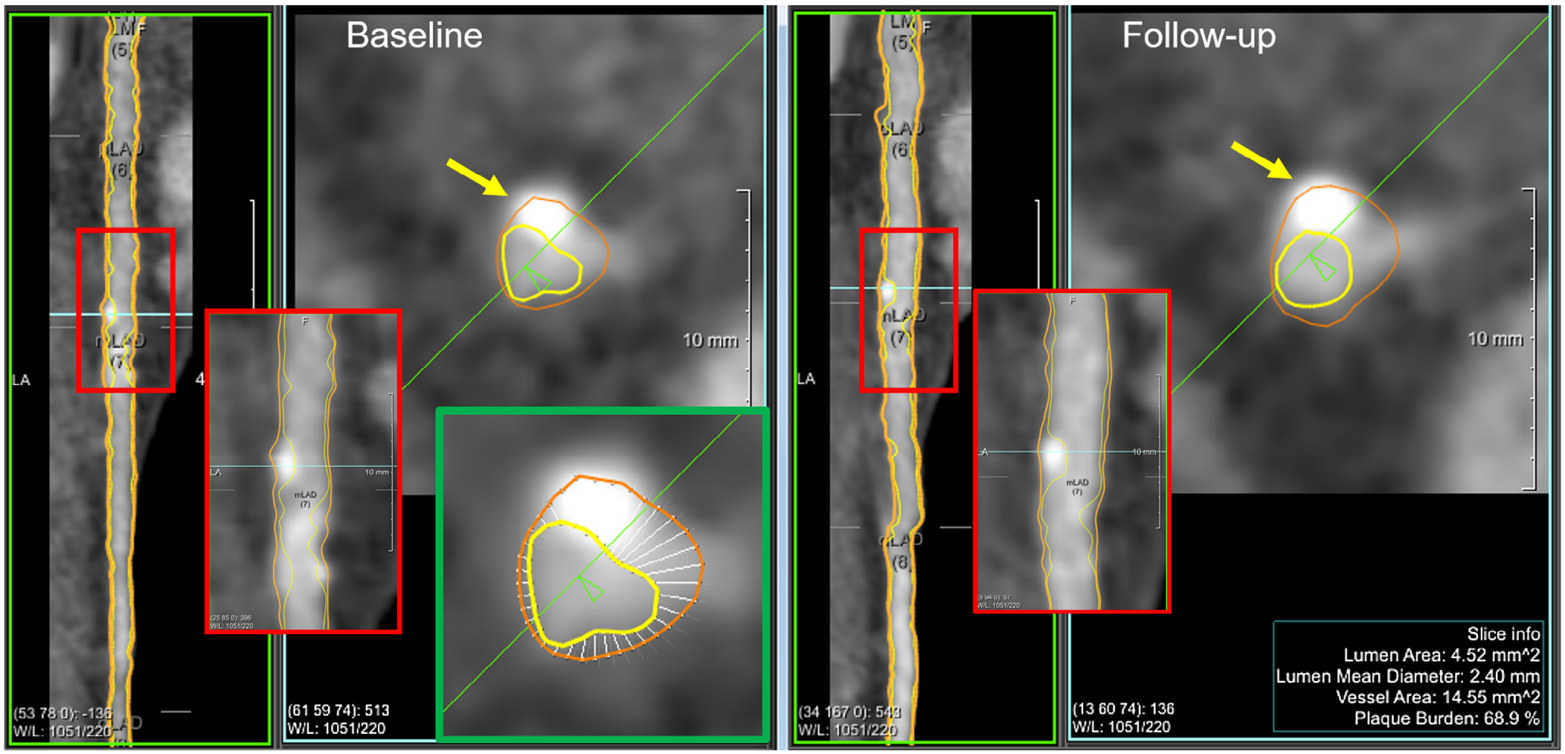
Traditional way of calculating the plaque thickness on a two‐dimensional (2D) slice and doing the baseline and the follow‐up plaque thickness comparison. Similar viewing angles were selected manually for assessing plaque changes in an artery between baseline and follow‐up; the red boxes include the location with coronary plaques and the close‐up image of the location; the yellow arrows point at the coronary plaque which is difficult to determine if the plaque changed. The viewing intensity window width and level are the same for the baseline and follow‐up images. The green box shows a close‐up image of calculating the plaque thickness on a 2D slice. The yellow color is the lumen contour and the red color is the vessel wall contour; the white straight lines are the distances between the lumen and the vessel wall contour measured every 10º around the lumen center. [Color figure can be viewed at http://wileyonlinelibrary.com]

Automated quantification of coronary plaques on CCTA images has become feasible due to the development of automatic extraction, labeling, quality assessment and lumen and vessel wall segmentation methods for coronary artery trees (CATs).^4^, ^5^, ^6^ Coronary lumen and vessel wall contours are detected in the multi‐plane reformatted images of the artery. Based on the detected lumen and vessel wall contours, the plaque thickness at a certain location in an artery can be calculated as the distance from lumen to the vessel wall (Fig. 1, the close‐up image in green box).

Plaque changes between a baseline coronary artery tree (*CAT‐BL*) and follow‐up coronary artery tree (*CAT‐FU*) are assessed on CCTA images to investigate the plaque development after a treatment, or to study the association with long‐term mortality.^7^, ^8^ Currently, plaque changes are measured manually as illustrated in Fig. 1. The corresponding arteries from the *CAT‐BL* and *CAT‐FU* are first visually assessed from a similar longitudinal viewing angle, and then aligned using anatomical landmarks, for instance, bifurcation points. Afterwards, coronary plaque differences are calculated based on the two‐dimensional (2D) transversal view and experts visually assess and grade the changes.^2^ However, manually selecting a viewing angle and landmarks for the alignment is time consuming and potentially introduces bias. It is difficult to decide whether the difference is a plaque change or caused by a slightly different viewing angle (Fig. 1, pointed out by yellow arrows). Moreover, calculating plaque changes in 2D does not utilize the three‐dimensional (3D) topology information.

Previous studies have demonstrated the feasibility of automatically registering 3D CATs from CCTA images^9^, ^10^, ^11^ which reduces manual alignment bias. However, most of these studies focused on registering CATs from different cardiac phases. Recently, Zeng et al.^10^ presented a method to align CATs from two time points but the changes in lumen and vessel wall were not considered.

To the best of our knowledge, automatic calculation of local changes in coronary plaque over time has not been studied in CCTA. Similar work on magnetic resonance imaging (MRI) images for automatically assessing local changes in carotid plaque morphology was presented by van't Klooster et al.^12^ However, compared to carotid arteries, coronary arteries are smaller in size and have more complex topologies which results in a more challenging task.

In this paper, we propose a novel method to automatically calculate and visualize the local changes in plaque thickness between *CAT‐BL* and *CAT‐FU*. The contribution of the manuscript is as follows,

1. The plaque thickness is calculated and shown on each location on the 3D surface of the coronary arteries instead of on the traditional 2D transversal slices.
2. A rigid registration method is used to match corresponding points on the baseline and follow‐up coronary surfaces.
3. Artificial baseline and follow‐up datasets are created to evaluate the proposed method in a well‐controlled environment. Multi‐center and multi‐vendor clinical datasets are used to evaluate the feasibility of the proposed method in clinical practice.

## 2 Materials and Methods

**Figure 2:**
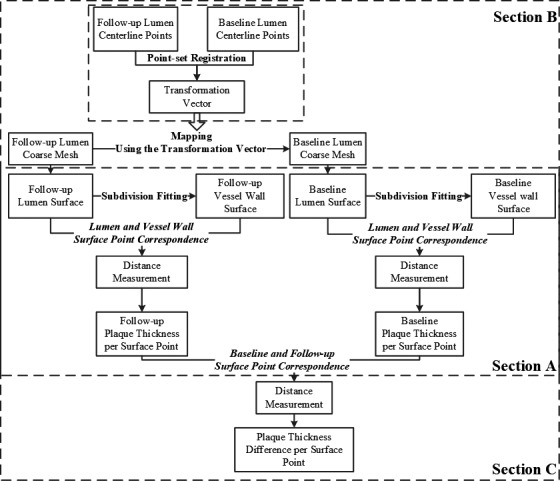
Workflow of the proposed method.

The workflow of the proposed automatic method for calculating the plaque thickness difference is shown in Fig. 2. We first calculate the plaque thickness for the *CAT‐FU* where the lumen and vessel wall are modeled as subdivision surfaces using the same coarse mesh. Then, the *CAT‐FU* coarse mesh is mapped to the *CAT‐BL* coordinate space using a transformation vector which is obtained from a point set registration on their lumen centerline points. Using the same coarse mesh, the *CAT‐BL* and *CAT‐FU* subdivision surfaces are created on which their plaque thicknesses are computed. In the end, the difference in the plaque thickness is calculated between corresponding *CAT‐BL* and *CAT‐FU* surface point. In the next section, we describe the creation of coronary subdivision surfaces.

### 2.A Plaque thickness on subdivision surface point

#### 2.A.1 Coronary subdivision surface

We use the so‐called “subdivision technique” to model the coronary lumen and vessel wall as smooth 3D surfaces. This technique allows to create a 3D surface from a coarse to a more fine‐grained mesh by repeatedly adding new vertices and edges according to certain subdivision rules.^13^ In this way a high‐resolution surface can be created from a low‐resolution initialization. Kitslaar et al.^14^ used the subdivision technique for coronary artery segmentation and showed the efficiency in modeling coronary arteries from CCTA images.

**Figure 3:**
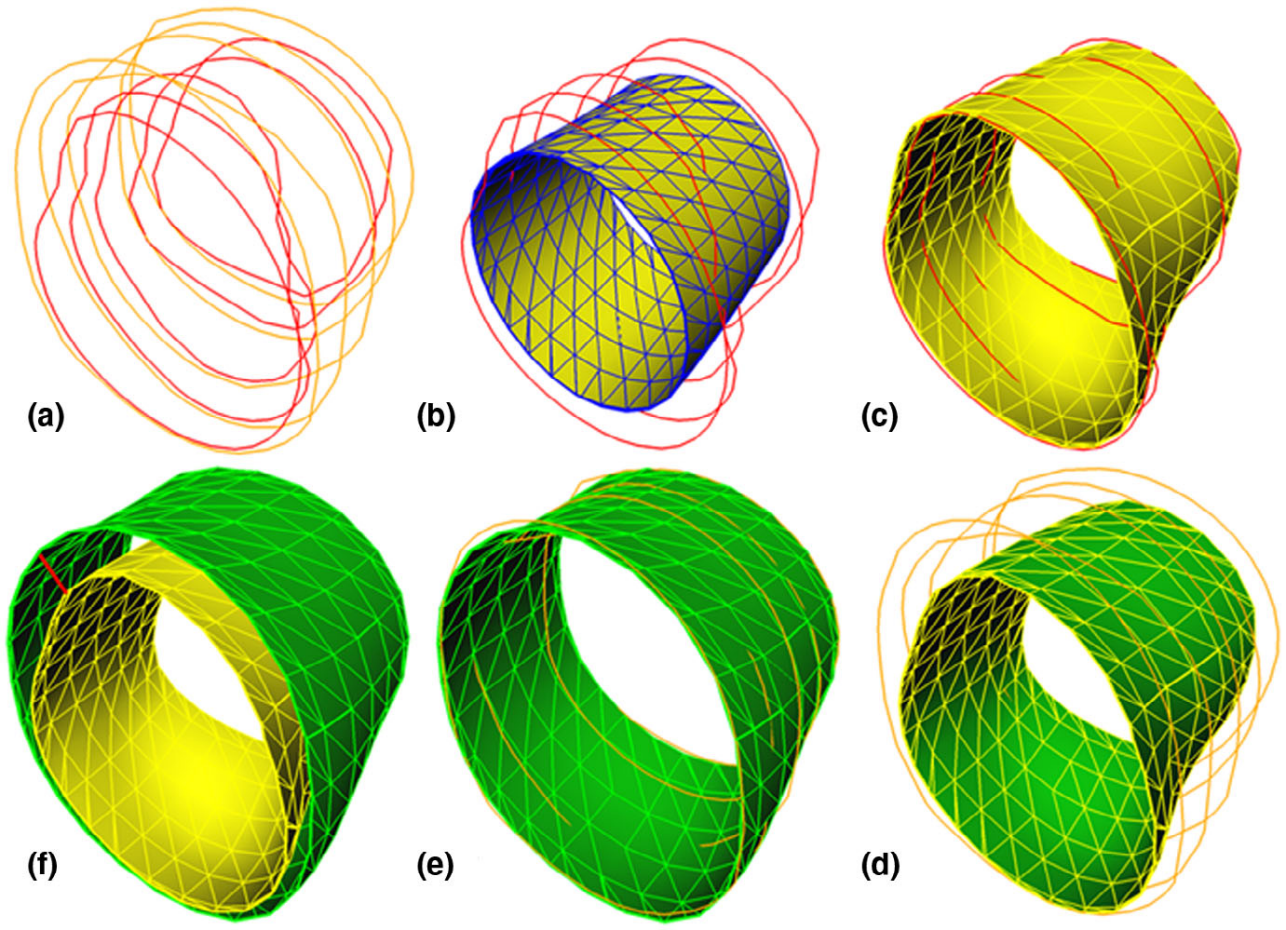
A process of generating coronary lumen and vessel wall surfaces by subdivision fitting to contours. From the top‐left subfigure to the bottom‐left subfigure in a clockwise direction are (a) lumen (red) and vessel wall contours (orange); (b) the initial lumen surface (blue) inside the lumen contours (red); (c) the final lumen surface (yellow) fitted to the lumen contours; (d) the initial vessel wall surface (green) inside the vessel wall contours (orange), and the initial vessel wall surface is actually the final lumen surface [the yellow surface in (c)]; (e) the final vessel wall surface (green) fitted to the vessel wall contours; (f) the plaque thickness (red straight line) on subdivision surfaces which is the distance between two mesh points on the final lumen and vessel wall surfaces. Legend: red = lumen contours, orange = vessel wall contours, blue = initial surface, yellow = lumen surface, green = vessel wall surface. [Color figure can be viewed at http://wileyonlinelibrary.com]

In our application, subdivision surfaces are initialized by hexagonal rings^13^, ^14^ that define a tubular shaped structure. The initial mesh will grow toward the target contours iteratively pushed by a force factor. The force factor indicates how a mesh point on the subdivision surface moves to the target contour, which is defined as the distance to the contour along the normal direction of the surface in the proposed method. Figure 3 shows an example process of generating coronary lumen and vessel wall surfaces by subdivision fitting to contours.

#### 2.A.2 Plaque thickness of a coronary artery tree

Similar to the traditional plaque thickness calculation on the contours (Fig. 1), we calculate the plaque thickness for a coronary subdivision surface point as the distance from the lumen surface point to the corresponding vessel wall surface point. Before we can calculate the plaque thickness, we need to find the correspondence between the lumen surface point and vessel wall surface point. In our method, the correspondence is obtained by using the same coarse mesh for both the lumen and vessel wall to initiate the subdivision fitting.

After the creation of the lumen subdivision surface (as described in the above section), we use it as the initialization for the vessel wall subdivision surface [Fig. 3(d)]. The lumen subdivision surface moves outwards from the lumen and fits to the vessel wall contours hereby generating the vessel wall subdivision surface [Fig. 3(e)]. In this way, the correspondence between the lumen and vessel wall surface points remains.

Finally, the plaque thickness for a lumen surface point is calculated as the distance to the corresponding vessel wall surface point which is depicted as a red straight line in Fig. 3(f).

### 2.B Baseline and follow‐up surface points correspondence

For the plaque thickness differences calculation between *CAT‐BL* and *CAT‐FU*, we first need to obtain the correspondence between the *CAT‐BL* and *CAT‐FU* surface points. Here, a similar approach as calculating the lumen and vessel wall surface point correspondence is adopted. We use the same coarse mesh to generate both *CAT‐BL* and *CAT‐FU* subdivision surfaces, so that only the mesh point position differs while their correspondence remains.

However, it should be realized that the *CAT‐BL* and *CAT‐FU* are in different coordinate spaces. We use a registration method to transform the coarse mesh between the *CAT‐BL* and the *CAT‐FU* coordinates. Here, we simplify the surface registration by first registering the centerline points and then using the obtained transformation vector to transform the surfaces.

#### 2.B.1 Coronary artery centerline points

Lumen centerline points are automatically detected from the coronary lumen contours using the method presented by Antiga et al.^15^ For all points on the extracted centerlines, a cubic spline interpolation is performed based on their absolute distances to the extracted ostia point and then down‐sampled to a point spacing of 0.5 mm.

#### 2.B.2 Coronary centerline points registration

In this work, we use the coherent point drift (CPD) point set registration method^16^ to register the centerline points from *CAT‐BL* and *CAT‐FU.* CPD registration has been widely applied in tree matching, and an important benefit of this method is that points are forced to move coherently as a group to preserve the topological structure of the point sets. The CPD algorithm contains both rigid and nonrigid point set registration schemes.

Considering the movements of the coronary arteries caused by the complex heart motion, nonrigid registrations were preferred by previous studies for matching CATs since it allows more elastic deformations.^9^, ^10^ CCTA images can be reconstructed from different phases of the cardiac cycle in which heart motion is involved resulting in shape deformations. Moreover, the extracted CATs can have different topologies, such as that some branches are missing in one of the CATs. Also, centerline points can shift between *CAT‐BL* and *CAT‐FU* due to the plaque size increase or decrease over time. However, these differences are treated as outliers or noise in the nonrigid CPD registrations which deteriorate the registration results and increase the registration time. Furthermore, nonrigid CPD allows the point spacing to change which increases the risk that points from different locations in the *CAT‐BL* and *CAT‐FU* are matched. After a comparison with the nonrigid registration, we use a rigid CPD registration in favor of the speed, robustness in noise and outliers, and point space preservation.

In the rigid CPD registration, we aim to align *CAT‐FU* and *CAT‐BL* using the following notations: *X*=(*x_1_,…,x_M_*)*^T^*, *Y*=(*y_1_,…,y_N_*)*^T^* which refers to points in *CAT‐FU* (the moving image) and *CAT‐BL* (the fixed image), respectively. *X* and *Y* are an *M*‐sized and a *N*‐sized vector of 3D points. *T*(*X*) represents the transformation vector for transforming *X* to match *Y*. After the rigid CPD registration, point correspondences are obtained. The obtained point correspondences are a *many‐to‐one* relationship which means that many points in the *CAT‐FU* (the moving image) could correspond to the same point in *CAT‐BL* (the fixed image). The *many‐to‐one* relationship is due to the potential difference in the number of points in the two point sets caused by the extraction. However, we want a *one‐to‐one* point correspondence to be able to do a precise comparison in a following procedure. Therefore, for a point *y_k_* in *CAT‐BL* which corresponds with one or more points from *CAT‐FU*, we find the closet match *x_i_* t and define the transformation vector as *T*(*x_i_*) = (*y_k_ − x_i_*). In this way, a *one‐to‐one* point correspondence between *CAT‐BL* and *CAT‐FU* is determined. We choose to transform the *CAT‐FU* to the *CAT‐BL* coordinates since a lumen decrease (plaque progression) in the *CAT‐FU* is more common than a lumen increase (plaque regression).

#### 2.B.3 Coronary artery surface mapping

To transform the *CAT‐FU* coarse mesh to the *CAT‐BL* space, we project the centerline points correspondence to the coarse mesh points. As was described in Section 2.A, coarse meshes are hexagonal rings along the centerline of a coronary artery, we use *x_i_^j^*, *j*∈[1,6], to denote the hexagonal ring of a centerline point *x_i_*. For two corresponding centerline points *x_i_* (in *CAT‐FU*) and *y_k_* (in *CAT‐BL*) with their transformation as *T*(*x_i_*) = (*y_k_ − x_i_*), we transform the hexagonal ring from *CAT‐FU* to *CAT‐BL* with *y_k_^j^ = T*(*x_i_*) + *x_i_^j^*. *y_k_^j^* is the transformed *x_i_^j^* in *CAT‐BL* coordinates, *j* ∈[1,6]. We perform the transformation for all hexagonal rings to achieve the coarse mesh transformation.

The transformed *CAT‐FU* coarse mesh is used as the coarse mesh for the subdivision fitting to get the *CAT‐BL* subdivision surfaces. During the subdivision fitting procedure, the point correspondence between the coarse mesh of the *CAT‐BL* and *CAT‐FU* remains. Hence, the correspondence between subdivision surface points on the *CAT‐BL* and *CAT‐FU* also remains.

### 2.C Plaque thickness difference

The plaque thickness is calculated for each lumen surface point of the *CAT‐BL* and *CAT‐FU* which are represented as *BL_Thic* and *FU_Thic*. The plaque thickness differences between corresponding *CAT‐BL* and *CAT‐FU* surface points are calculated as *DIF_thickness_*_ _= (*BL_Thic − FU_Thic*).

For visualization purposes, the plaque thickness differences are mapped to different colors to show the changes over time as shown in Fig. 6(a).

## 3 Experiments and Results

The proposed method was implemented as a module in MeVisLab 2.7.1 (MeVis Medical Solutions AG, Bremen, Germany). The experiments were performed on a PC, with a 2.67 GHz CPU, 12 GB memory and a 64‐bit Windows 10 system. The total procedure for calculating the plaque thickness difference between a coronary artery tree at baseline and at follow‐up can be finished within 5 min.

For the evaluation, we used both clinical scans and artificial datasets which were created based on clinical scans. We did the experiments to evaluate the points correspondence accuracy and the subdivision surface fitting accuracy. Furthermore, experiments were performed on clinical scans to assess the feasibility of the method in clinical practice.

### 3.A Datasets

In total, 12 multi‐center, multi‐vendor pairs of scans were used for the experiments, and we categorize them into three groups for different evaluation purposes. To enable the automatic coronary extraction and plaque comparison, the 12 pairs of scans used in this study were carefully checked by two experts that there were no large motion artifacts present that would interfere with a correct analysis. The lumen and vessel wall contours were extracted using the method presented by Boogers et al.^4^ and were manually corrected if needed by an expert.

To test the effect of different lumen and vessel wall contouring strategies, GROUP1 and GROUP2 were defined. GROUP1 contains five pairs of scans in which the vessel wall contours were always positioned outside the lumen contours, therefore resulting a thickness value always greater than zero. GROUP2 has three pairs of scans in which the vessel wall contours were positioned on the same location as the lumen contours when no plaques were present (resulting in zero thickness); If a plaque was present, the vessel wall contours were positioned outside the plaque. The follow‐up scans in GROUP1 and GROUP2 were done within an average period of 2 yr ± 1.6 months.

Finally, we defined GROUP3, consisting of four pairs of end‐of‐diastolic (*ED*) and end‐of‐systolic (*ES*) scans, in which the lumen and vessel wall contours were placed using the same rule as for GROUP1. The plaque thickness does not change between *ED* and *ES* scans which are used to assess the systematic bias of the proposed method. The Detailed information for the 12 pairs of scans can be found in Table 1.

**Table 1:** Datasets.

GROUP (number of scans)	Time point	Voxel size (x,y) (mm)	Slice thickness (mm)	Scanner list
1 (5)	BL	0.43 × 0.43	0.45	Brilliance 64
	FU	0.34 × 0.34		
2 (3)	BL	0.35 × 0.35	(0.25, 0.625)	LightSpeed VCT SOMATOM force SOMATOM definition AS Aquillion ONE
	FU	0.37 × 0.37		
3 (4)	ED and ES	0.45 × 0.45	0.625	Revolution CT

Note:

BL = baseline; FU = follow‐up; ED = end of diastolic; ES = end of Systolic. Voxel sizes are the average sizes of all data in one GROUP. For the slice thickness, the minimum and maximum values are presented when there are thickness differences within a group of the datasets. For some scans from GROUP2, the follow‐up scan was done with a different scanner from the baseline.

Additionally, one clinical scan was used to create 10 artificial pairs of *CAT‐BL* and *CAT‐FU*. The clinical scan is the first case in the training datasets (dataset00) from the MICCAI 2008 Coronary Artery Tracking Challenge with a voxel size of 0.32 × 0.32 × 0.4 mm.^17^

All the datasets in Group1 and Group2 were reconstructed at the mid‐to‐end diastolic phase (350 ms before the next R‐wave or at 65% to 70% of the R‐R interval). The *ES* and *ED* datasets in Group3 were reconstructed at the 45% and 75% of the R‐R interval, respectively.

### 3.B Points correspondence accuracy evaluation

**Figure 4:**
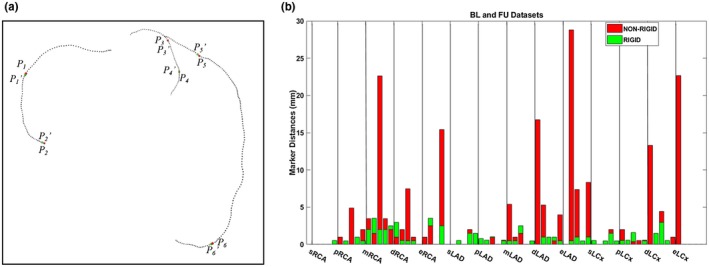
Distances between corresponding markers. (a) an example of markers on a *CAT‐BL* and the corresponding *CAT‐FU* markers after the registration. Markers on *CAT‐BL* (*p_i_*) and the registered markers (*p_i_^’^*) from *CAT‐FU* are denoted as red and green points, respectively. (b) Distances between markers on *CAT‐BL* and the registered markers from *CAT‐FU*. Y‐axis represents the calculated distances between registered markers. On x‐axis, markers are grouped by their positions on the artery. In each group, such as pRCA group, each bar represents the distance between two registered markers for one pair of datasets. Red bars denote the registered markers distances after nonrigid registration. Green bars denote the registered markers distances after rigid registration. s‐, p‐, m‐, d‐, e‐ represent the start, proximal, middle, distal, and end of the coronary artery on the bifurcation. RCA = right coronary artery; LAD = left anterior descending artery; LCx = left circumflex artery. BL = baseline; FU = follow‐up. [Color figure can be viewed at http://wileyonlinelibrary.com]

To evaluate the calculated points correspondence accuracy, centerline points were transformed using the obtained transformation vector and compared with ground truth correspondence. Therefore, markers were placed at the corresponding centerline points for both *CAT‐BL* and *CAT‐FU* by experts to create the ground truth. Corresponding markers were assigned to the start, proximal, middle, distal, and end parts of the arteries on bifurcation points. Expert 1 independently assigned all corresponding markers, and subsequently expert 2 checked the location and applied a correction if needed after a consensus reading with expert 1. Both experts were blinded to the registration results. Figure 4(a) shows an example of the six markers on a *CAT‐BL* (red points) and the corresponding *CAT‐FU* markers (green points) after the rigid registration. For each pair of markers, we calculated the Euclidean distance between the marker *p_i_* in *CAT‐BL* and the registered marker *p_i_^’^* in *CAT‐FU.* Furthermore, we compared rigid registration with nonrigid CPD registration for illustrating the advantages of using rigid CPD in this work.

For the point correspondence evaluation, nine pairs of scans (GROUP1 and GROUP3) were used. Since the markers were put in the start, proximal, middle, distal and end of the arteries and there are three main arteries in a coronary artery tree, an average of 14 markers were assigned to each pair of scans. In total 116 markers were included for the nine pairs of scans. In Fig. 4(b), each bar shows the distance for each pair of markers after the rigid (green) and nonrigid CPD registration (red) in GROUP1. The bars are divided into 14 groups based on the markers position using the black solid lines [Fig. 4(b)]. For example, the markers on mRCA, rigid registration shows better performance over the first, the third and the fourth case, whereas it has a slightly worse performance on the second and the fifth case. In general, regardless of the marker position, rigid CPD has a more stable and shorter distance over all 116 markers than the nonrigid CPD with the mean distance of 0.95 ± 0.98 mm vs 2.70 ± 5.10 mm, respectively. Specifically, rigid CPD has a slightly better performance on scans from GROUP1 (0.89 ± 0.92 mm) than GROUP3 (1.01 ± 1.04 mm).

### 3.C Evaluation on artificial pairs of datasets

A set of artificial datasets was created to evaluate the proposed method for calculating thickness changes for plaques with different sizes or plaques at different locations in the vessels. For the artificial datasets, the differences between the baseline and follow‐up were created mathematically by modifying the position of the lumen or vessel wall contours which resulted in known plaque thickness changes.

We use a clinical scan as *CAT‐BL* and manually edited its lumen or vessel contours to create a new CAT and then paired them up as *CAT‐BL* and *CAT‐FU.* In total, we created 10 artificial pairs of *CAT‐BL* and *CAT‐FU* datasets with a known plaque thickness. For evaluating the ability to measure plaque changes, we decreased the lumen radius with different sizes and different lengths along the vessel direction to simulate different degree of plaque progression. Similarly, we increased the lumen radius to simulate plaque regression. To simulate the plaque positive remodeling, we increased the vessel wall radius, whereas the lumen radius remained unchanged. We put these created plaques at different locations in a coronary artery and in different arteries to determine the robustness in measuring plaque changes at different locations. Furthermore, the lumen and vessel wall radius were increased with the same size to simulate the lumen increase with no change in plaque thickness. Details of the 10 artificial datasets can be found in Table 2.

**Table 2:** Datasets description and results for artificial datasets and end‐of‐diastolic (ED) — end‐of‐systolic (ES) scans.

No.	Variable	Simulation	Created DIFthickness (mm)	Calculated average DIFthickness (mm)
1	*L* = 4.00 mm *∆R_lumen _*= − 0.50 mm	Plaque increase with different lengths	+0.50	0.42 ± 0.08
2	*L* = 6.00 mm *∆R_lumen _*= − 0.50 mm		+0.50	0.45 ± 0.05
3	*L* = 2.00 mm *∆R_lumen _*= − 0.50 mm		+0.50	0.41 ± 0.05
4	*L* = 4.00 mm *∆R_lumen _*= − 0.25 mm	Plaque thickness increase with differences in transversal direction	+0.25	0.21 ± 0.09
5	*L* = 4.00 mm *∆R_lumen_* = − 1.00 mm		+1.00	0.88 ± 0.17
6	*L* = 4.00 mm *∆R_lumen_* = + 0.5 mm	Plaque thickness decrease in transversal direction	−0.50	−0.46 ± 0.06
7	*L* = 4.00 mm *N_plaque_* = 3	Plaque thickness increase on the proximal, middle and distal part of an artery	+0.50	0.42 ± 0.08 (proximal) 0.43 ± 0.07 (middle) 0.44 ± 0.06 (distal)
8	*L* = 4.00 mm *N_plaque_* = 3	Plaque thickness increase on the RCA, LAD and LCx	+0.50	0.42 ± 0.08 (RCA) 0.43 ± 0.07 (LAD) 0.43 ± 0.07 (LCx)
9	*L* = 4.00 mm *∆R_vessel _*= + 0.50 mm	Plaque increase with vessel wall size increase	+0.50	0.43 ± 0.09
10	*L* = 4.00 mm *∆R_lumen _*= + 0.50 mm *∆R_vessel _*= + 0.50 mm	Increase both lumen and vessel wall with plaque thickness difference as 0	0.00	−0.00 ± 0.03
ED and ES‐01			0.00	−0.02 ± 0.21
ED and ES‐02			0.00	−0.01 ± 0.21
ED and ES‐03			0.00	0.02 ± 0.15
ED and ES‐04			0.00	−0.02 ± 0.16

Note:

*L* = length of a plaque along the vessel direction; *N_plaque_* = number of plaques; *DIF_thickness_* = difference in plaque thickness; *∆R_lumen_* = lumen increase/decrease radius; *∆R_vessel_* = vessel wall increase/decrease radius; RCA = right coronary artery; LAD = left anterior descending artery; LCx = left circumflex artery; ED = end of diastolic; ES = end of systolic.

For the 10 pairs of artificial datasets, the proposed method successfully found plaque thickness differences between *CAT‐BL* and *CAT‐FU* datasets. In general, on the location where we created the plaque thickness difference, the average of the calculated plaque thickness difference is the same as the ground truth value with the standard deviation (SD) of ±0.10 mm. For cases where we created plaque increase with different lengths (No. 1, 2 and 3), the calculated average *DIF_thickness_* is 0.42 ± 0.06 mm. Varying position of the plaque (Nos. 7 and 8) still results in the calculated average *DIF_thickness_ = *0.43 ± 0.07 mm. For case No. 9 where the expected *DIF_thickness_* = 0.00 mm, the proposed method calculated the average plaque thickness difference as 0.00 mm with the minimum and maximum plaque thickness as −0.08 and 0.13 mm, respectively. Details of the calculated plaque thickness differences for each pair are listed in the last column of Table 2.

**Figure 5:**
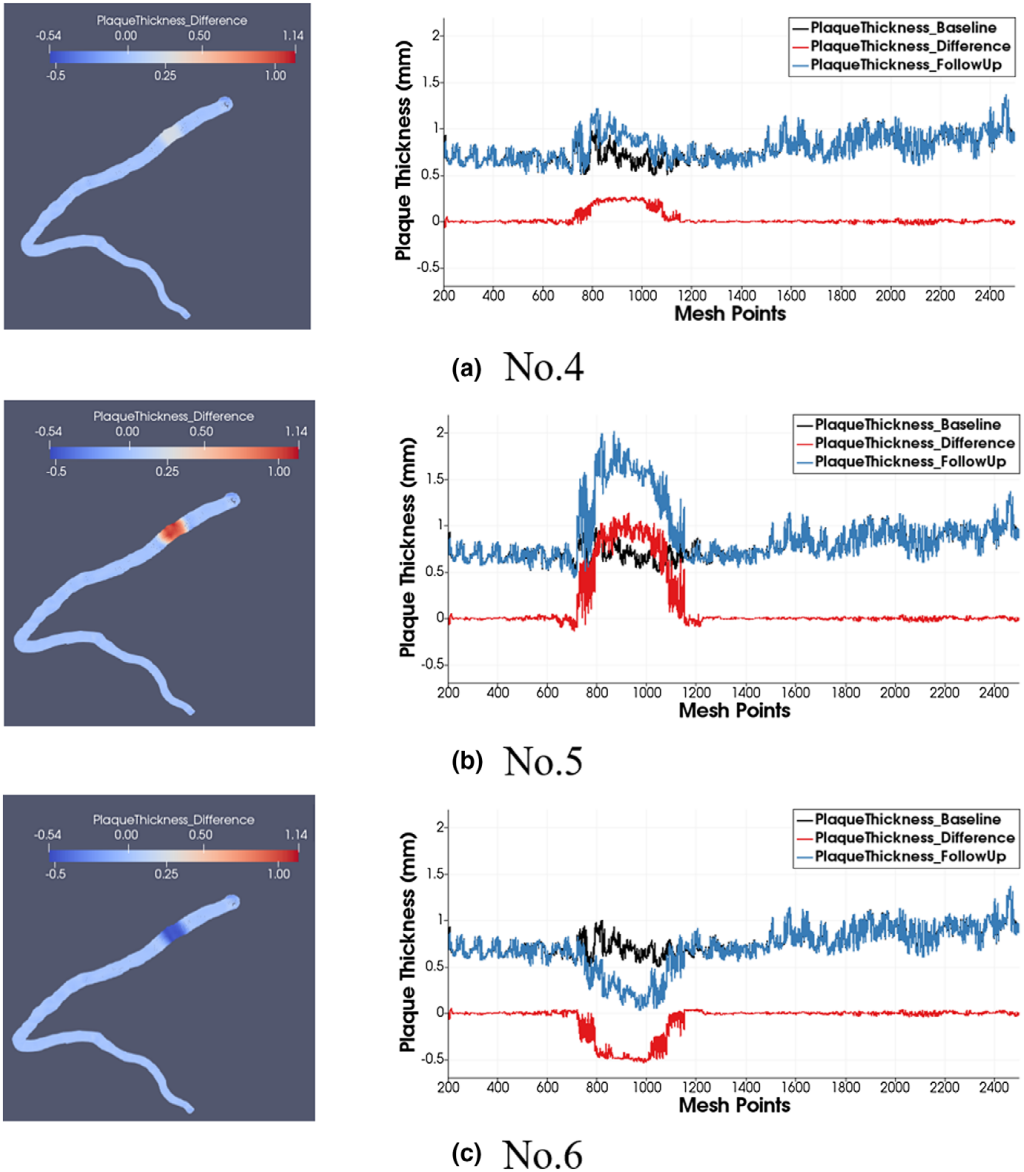
Plaque thickness calculated by the proposed method for artificially created baseline and follow‐up pairs for No. 4, 5, and 6 in Table 2 with their average plaque thickness as 0.21 ± 0.09 mm, 0.88 ± 0.17 mm, and −0.46 ± 0.06 mm, respectively. The left column is the measured plaque thickness difference with color coding on the modeled coronary subdivision surfaces, and the right column shows the plaque thickness for baseline, follow‐up and their differences for each mesh point along this artery. [Color figure can be viewed at http://wileyonlinelibrary.com]

Figure 5 shows examples of calculated plaque thickness differences for case Nos. 4, 5, and 6 which represent 0.25 mm increase, 1.00 mm increase, and 0.50 mm decrease of plaque thickness, respectively. For all mesh points along the artery (x‐axis), the proposed method located the plaque changes exactly at the targeted position. The average of the measured *BL_Thic*, *FU_Thic,* and *DIF_thickness_* (y‐axis) are the same as the corresponding manually created values.

### 3.D Plaque thickness differences on clinical scans

For 12 pairs of clinical scans (GROUP1, −2, and −3), the proposed method was performed to calculate the differences in plaque thickness for each surface point.

**Figure 6:**
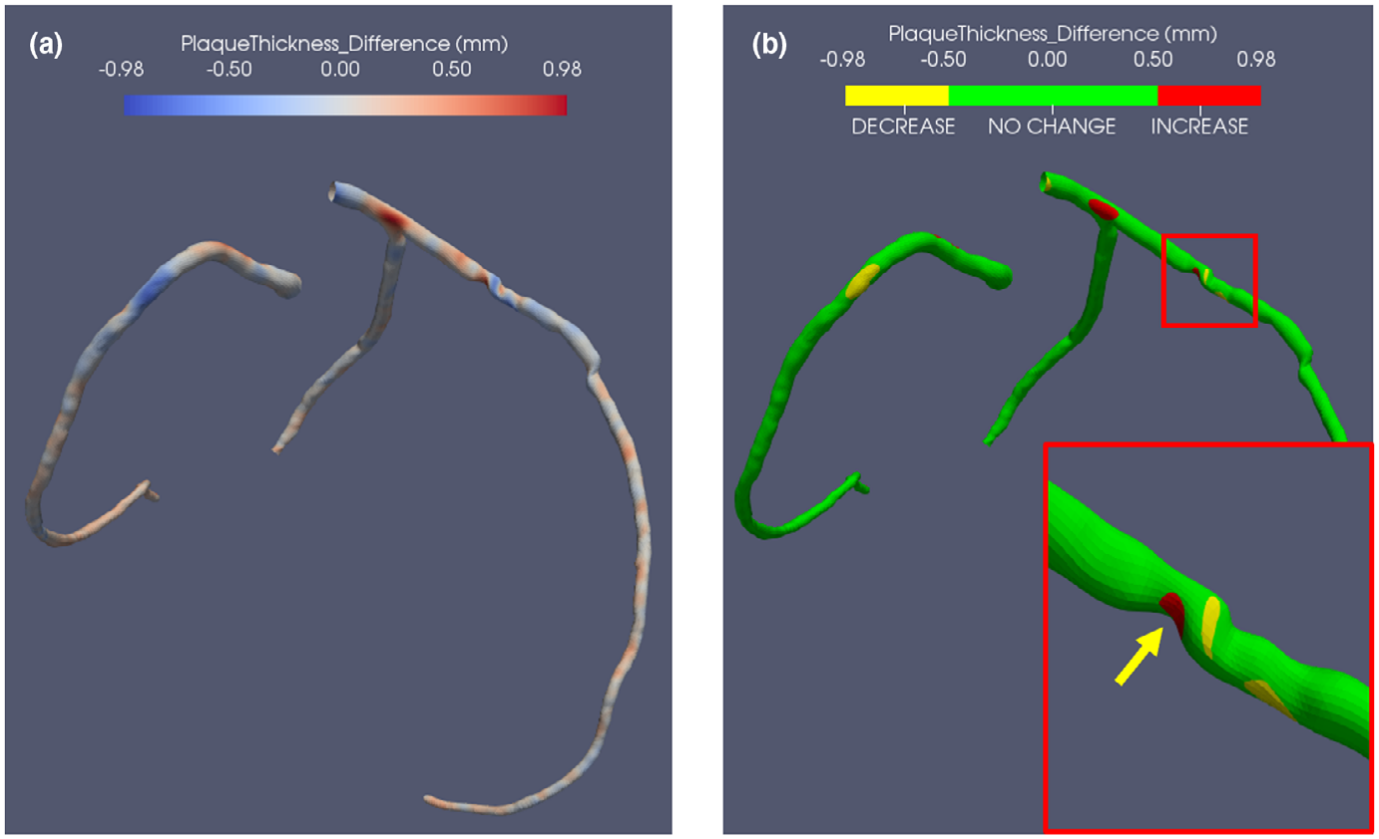
The proposed way of baseline and follow‐up plaque thickness difference assessment and the automatic quantification of plaque thickness changes. (a) The automatically calculated plaque thickness changes with color coding on coronary subdivision lumen surface points. (b) The three‐dimensional visualization of large plaque thickness increase/decrease using a threshold 0.5 mm; The red box includes the close‐up image of the plaques with the yellow arrow pointing at the same plaque as shown in Fig. 1. [Color figure can be viewed at http://wileyonlinelibrary.com]

Figure 6 shows an overview of the baseline and follow‐up comparison for a case from GROUP1 using the proposed method. On Figs. 6(a) and 6(b), 3D views of the plaque thickness changes between baseline and follow‐up are presented. For this case from GROUP1, the automatic results by the presented method showed that there is a plaque thickness change at every location of the CAT [seen from Fig. 6(a)] which is caused by the manual bias during the delineating of the lumen and vessel wall contours for *CAT‐BL* and *CAT‐FU*. For a better visualization purpose, a threshold (||0.5|| mm) is selected as a cut‐off value to focus on large plaque thickness changes. On Fig. 6(b), the calculated *DIF_thickness_* in the range [−0.5 mm, 0.5 mm] is denoted as no plaque change. The calculated *DIF_thickness_ *< −0.5 mm is described as a decrease of a plaque, whereas the calculated *DIF_thickness_ *> 0.5 mm is described as an increase of a plaque. The plaque increase is denoted by red and the plaque decrease is denoted by yellow which provides a straightforward overview for the users [Fig. 6(b)].

**Figure 7:**
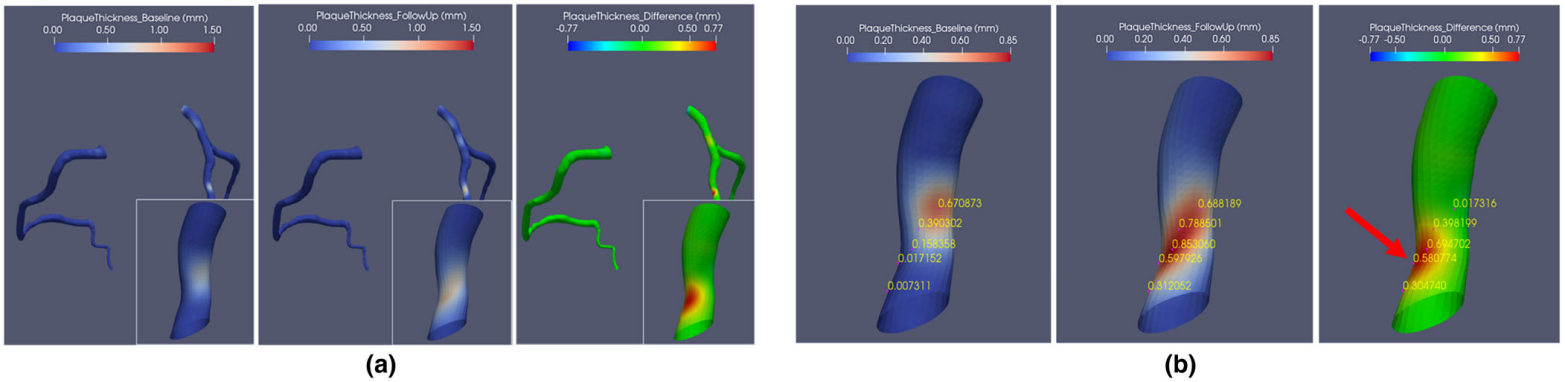
Three‐dimensional coronary subdivision surfaces with the plaque thickness for baseline, follow‐up and their differences color coded. (a) An overview of a case for the plaque thickness at baseline, follow‐up and their differences; (b) Close‐up images of the plaque in (a), on corresponding mesh points, the plaque thickness values were assigned. The red arrow points at the position where there is a plaque size increase along the coronary blood stream [Color figure can be viewed at http://wileyonlinelibrary.com]

Figure 7 demonstrates the measured plaque thickness differences between *CAT‐BL* and *CAT‐FU* for a scan from GROUP2. In total, for 27867 mesh points on the generated *CAT‐BL* and *CAT‐FU* subdivision surfaces, the minimum and maximum for *BL_Thic* and *FU_Thic* are 0.00 and 0.85 mm, respectively. The measured minimum and maximum *DIF_thickness_* are −0.57 and 0.77 mm, respectively. In Fig. 7(b), close‐up images for a plaque are shown where the thickness values for corresponding mesh points on *CAT‐BL*, *CAT‐FU* and the plaque thickness differences images are highlighted. As can be seen from Fig. 7(b), there was a plaque in *CAT‐BL*, and the plaque is still present at *CAT‐FU*, whereas the plaque size increased on a location (pointed by a red arrow) distally along the blood stream direction.

For the *ED* and *ES* scans from GROUP3 in which the *DIF_thickness_* = 0.00 mm, the average of the calculated *DIF_thickness_* is −0.00 mm with a SD = ±0.18 mm. Values of the calculated plaque thickness differences for each pair of the four scans in GROUP3 can be found in Table 2.

## 4 Discussion

In this paper, a method to automatically calculate local plaque thickness differences between a coronary artery tree on baseline and follow‐up CCTA images is presented.

A rigid CPD registration was used in this work due to its high accuracy and robustness in coronary trees acquired at two time points (*CAT‐BL* and *CAT‐FU*) or two phases (*ED* and *ES*). Even though nonrigid CPD registration allows more deformations which could find an exact match for each point, the purpose of this work is to find a list of *one‐to‐one* corresponding points without changing the centerline point spacing. By first doing a rigid CPD registration and then calculating the closest point, we are able to find corresponding points with the local changes between two CATs preserved. According to the experiments, the proposed method generates points correspondence within 1 mm deviation which is within two voxels. The registration performed slightly worse on scans from two cardiac phases than scans from a baseline and follow‐up which is probably due to more coronary shape deformations between scans from two phases. The matching results for the distal part of coronaries are slightly less accurate compared to the proximal parts. It could be caused by differences in the extraction of distal parts due to less contrast in distal part of the artery. From a clinical point of view, plaques in the distal part of a coronary artery are not that relevant for clinical decision making.

We find the surface point correspondence of two CATs by subdivision fitting with the same coarse meshes. The *CAT‐FU* coarse meshes are transformed to *CAT‐BL* coordinates and used as the coarse mesh for *CAT‐BL*. With the same coarse meshes as an initialization for the subdivision fitting, the mesh point correspondences were obtained directly. We choose to transfer *CAT‐FU* to *CAT‐BL* since plaque progression is more common than plaque regression, resulting in a smaller lumen in a follow‐up CAT which fits our idea of creating a coarse mesh entirely inside of the lumen. However, the subdivision fitting is also able to grow towards the inside of the lumen which means transforming *CAT‐BL* to *CAT‐FU* should not affect the final calculation of the plaque thickness.

Our experiments on artificially created *CAT‐BL* and *CAT‐FU* pairs showed that the calculated plaque thickness has an average deviation of 0.10 mm from the expected values. The deviation is caused by the approximation of the subdivision fitting process for modeling the coronary surfaces. Furthermore, it should be noted that potential motion artifacts could still exist in the *CATs* but these do not have a big influence on the final results. Accordingly, the average of the calculated plaque thickness differences for *ED* and *ES* scans is zero with a SD of 0.18 mm which could be caused by the lumen and vessel wall contour delineation, the registration, the surface subdivision fitting and potential motion artifacts.

For the visualization purpose, we used a fixed threshold as a cut‐off value to focus on the large plaque thickness increase and decrease in Fig. 6(b). The threshold (0.5 mm) is obtained based on the calculated plaque thickness differences of all scans in GROUP1 which is related to the delineation of the lumen and vessel wall contours. From the calculation for artificial datasets (SD ± 0.10 mm) and *ED* and *ES* scans (SD ± 0.18 mm), the systematic bias was about 0.18 mm. Therefore, the threshold should be large enough to be able to visualize plaque changes. To determine the plaque progression or regression, a smaller value can be used depending on the presence of a systematic bias. Furthermore, the systematic bias of the whole process was about 0.18 mm which is half of the voxel size of the CCTA images.

To the best of our knowledge, the current tools in PACS calculate the plaque thickness on 2D slices the same as illustrated by the Fig. 1 (the close‐up image in green box) which could be performed in a few seconds. The comparison of the plaque thickness between the baseline and the follow‐up is performed manually by selecting an artery, and then the corresponding slice, which is shown in Fig. 1. However, this process could make the calculation nonreproducible and could take very long time to manually find the corresponding location and viewing angle in 2D slice. The proposed method calculates the plaque thickness at multiple locations in multiple slices for the entire coronary arteries automatically and the calculation is reproducible.

### 4.A Limitations

The proposed method relies on the delineated lumen and vessel wall contours which in this method were first created by the automatic software and afterwards corrected by experts.

## 5 Conclusions

The presented automatic plaque thickness comparison method can successfully locate, calculate and visualize the changes in coronary plaque thickness over time for a specific location in the coronary artery. This will benefit the automatic analysis and reporting of the coronary plaque progression and regression.

## Conflicts of Interest

Pieter Kitslaar is employed by Medis medical imaging systems bv and has a research appointment at the Leiden University Medical Center. Mingyuan Yuan is employed by Zhoupu Hospital, Shanghai university of medicine & health Science, China and have provided three scans for the experiments in the manuscript. The remaining authors have no conflicts of interest to disclose.
